# Human Milk for Vulnerable Infants: Breastfeeding and Milk Sharing Practice among Ghanaian Women

**DOI:** 10.3390/ijerph192416560

**Published:** 2022-12-09

**Authors:** Cecilia Obeng, Frederica Jackson, Christiana Nsiah-Asamoah, Salome Amissah-Essel, Barnabas Obeng-Gyasi, Cydne A. Perry, Ines Gonzalez Casanova

**Affiliations:** 1 Department of Applied Health Science, School of Public Heath, Indiana University, Bloomington, IN 47405, USA; 2 Department of Clinical Nutrition and Dietetics, University of Cape Coast, Cape Coast TF0494, Ghana; 3 Department of Health, Physical Education and Recreation, University of Cape Coast, Cape Coast TF0494, Ghana

**Keywords:** breastfeeding, milk sharing, milk donation, human milk banks, infant health

## Abstract

Human milk has the best impact on childhood survival. In Ghana, it is estimated that 43% of women exclusively breastfeed for 0–5 months and only 42% of breastfeeding mothers continue through 20–23 months. Although the Ghanaian government has implemented policies to facilitate exclusive breastfeeding, substantial gaps to achieve optimal newborn health and wellbeing remain. The purpose of this study was to evaluate breastfeeding prevalence and human milk sharing practices among Ghanaian women. Qualitative responses were received from Ghanaian females (n = 1050). In our sample, 81% indicated they breastfed their children and 8% reported ever sharing breastmilk with another mother. Reasons for sharing milk included (i) insufficient breastmilk production of the recipient mother, and (ii) mother’s unavailability prompting women to offer their milk to a crying baby. About 60% of our sample reported that they were not concerned about sharing their milk. Findings present a strong indicator for milk donation towards the establishment of a human milk bank in Ghana. Health promotion efforts should aim at increasing education about the risks involved in milk sharing as well as the benefits of human milk donation through formal and safer channels such as a Human Milk Bank.

## 1. Introduction

Malnutrition contributes to early childhood deaths as it increases children’s vulnerability to diseases [1]. Breastmilk has been identified as the ideal nutrition source to prevent infant morbidity and mortality but several factors may prevent mothers from breastfeeding their infants.

Ghana has a high rate of infant mortality. In 2020, infant mortality in Ghana was projected at 33 deaths per 1000 live births [2]. Neonatal mortality is equally high and was estimated at 23 deaths per 1000 live births in 2020 [2]. Overall, under-five mortality rates have dropped significantly in the past 3 decades from 127.4/1000 in 1990 to 44.7/1000 in 2020 [2]; however, there is still room for improvement. Regarding breastfeeding rates, according to the most recent UNICEF data, the percentage of exclusive breastfeeding for 0–5 months among Ghanaian women is 43% while continued breastfeeding through 20–23 months is estimated at 42% [2]. The government of Ghana has implemented several policies to facilitate exclusive breastfeeding practices including paid maternity leave for up 12 weeks post-delivery and hourly breaks for nursing mothers to breastfeed during working hours [3]. Besides breastfeeding support, Ghana has executed numerous maternal and child health programs that are focused on reducing maternal and infant mortality rates in the country. While these programs are helping to improve health outcomes for both mother and child, there are still substantial gaps to achieving optimal health and wellbeing for this population, especially for newborns. For example, major gaps in breastfeeding counselling and care for HIV-positive mothers have been identified [4]. Additionally, existing evidence shows a continuous decline in breastfeeding indicators (early initiation of breastfeeding within one hour of birth, exclusive breastfeeding for first six months of life and breastfeeding until two years). Reports from previously conducted Demographic Health Surveys indicate that whereas in 2008 exclusive breastfeeding rate in Ghana was 62%, it dropped to 53% in 2014 [5]. The Multiple Indicator Cluster Survey (MICS2017/18) revealed a further decline (42.9%) in exclusive breastfeeding rates in Ghana [6].

A closer look at exclusive breastfeeding rates in the country reveals inconsistencies and wider gaps. For instance, a study by Appiah et al. (2021) investigating breastfeeding practices in the volta region of Ghana among a sample of 396 mothers found that only 61% initiated breastfeeding within one hour of birth [7]. Additionally, 5.1% gave fluids besides breastmilk to the baby on the first day. Interestingly, 11% of study participants had never heard of exclusive breastfeeding and only 43.7% were practicing exclusive breastfeeding at the time of the study [7]. Moreover, a study carried out in Ghana revealed that infants of normal birth weight and high birth weight were more likely to be exclusively breastfed compared to low-birthweight infants. Likewise, infants assumed to be of normal birth size were more likely to be exclusively breastfed compared to infants perceived to be of small birth size [8]. Similar findings have been reported in other developing countries [9,10,11,12]. These findings underscore the need to adopt additional strategies and supportive care interventions to promote early initiation of breastfeeding and exclusive breastfeeding especially for vulnerable newly born babies (preterm, caesarean section-delivered, low-birthweight). One intervention that has been strongly advocated for and is currently being explored is the provision of human milk from donor lactating mothers.

Donor human milk has the potential to fill gaps when breastmilk is not available and to support exclusive breastfeeding. Donated human milk can promote exclusive breastfeeding for low birthweight babies who are admitted at the neonatal intensive care unit (NICU) for extended time periods. While it is recommended that mothers continue to facilitate close contact (rooming-in) after delivery, which is reported to stimulate milk production, this process is interrupted by long NICU stays for low birthweight and preterm infants. Similarly, women who undergo emergency caesarean sections (c-sections) have been found to have a higher proportion of breastfeeding difficulties, when compared to those who delivered vaginally or opted for and planned for c-sections [12,13,14,15]; they are more likely to discontinue breastfeeding before 12 weeks postpartum compared to women who delivered vaginally [12]. In these cases, donated milk can be ideal to support the initiation and continuation of breastfeeding, particularly for women experiencing delayed milk production and undersupply.

A limitation of human milk donation initiatives, especially in low- resource settings like Ghana, is that there is paucity of information regarding mothers’ motivation for sharing breastmilk and potential concerns regarding the practice of accepting milk from other lactating mothers. The current study therefore investigates breastfeeding and milk sharing practice among Ghanaian women and its impact on the health of children.

## 2. Materials and Methods

Following approval by Indiana University’s Institutional Review Board (ethical approval number 15224) in April 2022, recruitment and data collection procedures were initiated. We used a combination of web-based and face-to-face recruitment approaches. Specifically, about 80% (1016) of our sample was recruited via social media (Facebook group ‘Tell It Moms’, an all-female Ghanaian support group). An anonymous link to the survey was developed using Qualtrics survey software and was distributed on the social media platform. Additional respondents were recruited face-to-face within the Ghanaian community through word-of-mouth and subsequently using the snowball sampling technique. Aspects of Babchuk’s (2019) steps for guiding qualitative data analysis were used [16]. The steps used in this research included the coders: (1) refamiliarizing themselves with the narratives in the data, (2) initially using open coding procedures (constantly comparing similar narratives in the data), (3) constructing categories and assigning codes to them, (4) the two coders creating themes from categories independently. An experienced qualitative researcher was recruited to verify that the themes selected by the coders were true representation of the data. The results of the study are explained below.

## 3. Results

The socio-demographic characteristics of the women who participated in the study is presented in Table 1.

**Figure 1:**
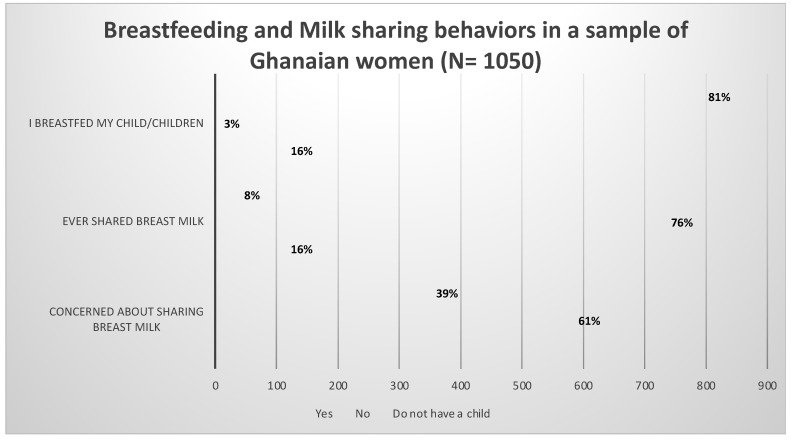
Breastfeeding and milk sharing behaviors in a sample of Ghanaian women.

Respondents were asked to indicate if they breastfed their child(ren). Again, participants were asked if they had ever shared breast milk and whether they had concerns about sharing their milk. The results are shown in Figure 1.

Out of the 1050 women who participated in the study, 853 (81%) indicated they breastfed their children, 25 (3%) reported they did not breastfeed their children and 172 (16%) indicated they did not have children. Further, 86 (8%) respondents indicated they had previously shared their breastmilk with another mother while 792 (76%) had never shared milk. Meanwhile, 641 women (61%) reported that they were not concerned about sharing their breastmilk.

### 3.1. Qualitative Results

The results which were generated from six questions are presented and elucidated in this section. A summary of study questions, themes, subthemes and participant quotes are provided in Appendix A (Table A1) of this manuscript.

The questions dealt with:

- Why mothers breastfeed their children;
- Why mothers chose not to breastfeed their children;
- Mothers motivation for sharing their breast milk with other women/mothers;
- Mothers motivation for not sharing breast milk with other women/mothers;
- Having concerns about sharing breast milk; and
- Having no concerns about sharing breast milk

#### Reasons for Breastfeeding

We begin with the question that dealt with why mothers breastfed their children.

A. Why Mothers Breastfeed Their Children

To the question, you indicated that you breastfed your child(ren), explain why you chose to breastfeed, five hundred and seventy-seven (577) participants responded and two themes emerged as reasons why the respondents chose to breastfeed. The themes are: “Ideal for child development” and “Affordable.”

1. Breast Milk Being Ideal for Child Development

Respondents explained that they breastfed their child because of the numerous nutritional benefits and its contribution to the growth and development of babies. This is illustrated in the following quotes:

Excerpt 1
Breast milk is very vital and ideal for child development. It contains all the nutrients babies need to grow. (Currently unemployed, 30 years, resident in Southern part of Ghana)
Excerpt 2
Because it helps in good child development and also prevent infections. (Public sector worker, 27 years, resident in Southern part of Ghana

2. Breast Milk is Affordable

Excerpt 3
Because it is cheaper and a better option. (Private sector worker, 30 years, resident in Southern part of Ghana)
Excerpt 4
Breast milk contains the right nutrients for babies, and it is cheap and readily available. (Public sector worker, 34 years, resident in Southern part of Ghana)

B. Why Mothers Chose Not to Breastfeed Their Children

Only six (6) respondents of 25 answered the question regarding why they chose not to breastfeed their infant. The primary reason for not breastfeeding was “insufficient breastmilk”. Excerpt 5 illustrates this finding.

Excerpt 5
I did not have sufficient breast milk (Private sector worker, 27 years, resident in Southern part of Ghana)

C. Motivation for Sharing Breast Milk with Other Women

Thirty-two (32) respondents answered the question concerning what motivated them to share their breast milk with other lactating mothers. Two themes emerged from the participants’ responses: “Insufficient breast milk production” and “Mother not available.” Each theme is exemplified below.

1. Insufficient Breast Milk Production by Infants’ Mothers

Most respondents indicated that the lack of adequate breast milk production of other mothers prompted them to share their breast milk. This is illustrated in following quotes:

Excerpt 6
My baby refused to suckle, and the other woman saw me expressing and discarding my breast milk so she came, pleading to give it to her instead because she had inverted nipples and also couldn’t produce breast milk and her baby was reducing in weight because she could not afford formula. (Public sector worker, above 35years, resident in Northern part of Ghana)
Excerpt 7
I was engorged. And she wasn’t producing enough to feed her child but was determined to stick to the 6 months rule. (Public sector worker, 31years, resident in Southern part of Ghana).

2. Mothers Unavailability Prompting women to Share their Breast Milk

Some respondents reported that the absence of an infant’s mother prompted them to share their breast milk. They shared the following quotes:

Excerpt 8
The baby was same month as mine. His mother had to go somewhere and return quickly but something delayed her, and baby started crying so I breastfed him. (Currently unemployed, 30years, resident in Southern part of Ghana).
Excerpt 9
Her breast milk was initially not flowing and later [the mother] was admitted at the hospital because of complications. (Public sector worker, 31 years, resident in Southern part of Ghana).

D. Motivation for Not Sharing Breast Milk with Other Women

Six hundred and sixty-one (661) respondents answered the question regarding why they did not or were not motivated to share their breast milk with other mothers/women. Four themes were identified. The themes are: “No opportunity”, “Not screened”, “Did not have enough” and “Personal perception.” Each of these are elucidated and exemplified below.

1. Absence or Lack of Opportunity to Share Breast Milk

Respondents explained that they had not shared breast milk because they had not had the opportunity to do so in the past. Extracts 10 and 11 exemplify and help give credence to the above assertion.

Excerpt 10
No opportunity to do so.
(Public sector worker, 27 years, resident in Southern part of Ghana).
Excerpt 11
I produce enough and haven’t had the opportunity to share.
(Public sector worker, 36 years, resident in Southern part of Ghana).

2. Not Screened

Some of the respondents felt that they needed to be screened before sharing their breast milk. The quote below illustrates this point.

Excerpt 12
Well, I know that one need to be screen before and certified before sharing her breast milk to other mothers, so I believe am not fit until I get screened. (Public sector worker, 38 years, resident in Southern part of Ghana)
Because no screening has been done and we know breast milk can transfer infections.
(Public sector worker, 40 years, resident in Southern part of Ghana).

3. Insufficient supply of Breast Milk

Some of the respondents explained that they were not able to share their breastmilk because they experienced undersupply themselves. Excerpts 13 and 14 are cited below in support of this theme.

Excerpt 13
Unfortunate I did not have enough breast milk myself, aside that I do not know any other breastfeeding mom who was in dire need of breast milk.
(Public sector worker, 36 years, resident in Southern part of Ghana).
Excerpt 14
I seldom have enough to spare.
(Public sector worker, 44 years, resident in Southern part of Ghana).

4. Personal Perceptions about Breast Milk Sharing

Some of the respondents gave their personal thoughts and experiences about sharing their breast milk with others. The following quotes illustrate this point:

Excerpt 15
Because only my baby needs my breast milk, and I don’t think someone in Ghana will allow her baby to be fed by a person who isn’t even a relative.
(Public sector worker, 31 years, resident in Southern part of Ghana).
Excerpt 16
Because they feel some way when you offer it [breast milk] to them… I have offered it before, but I was turned down.
(Public sector worker, 27 years, resident in Northern part of Ghana).

E. Concerned about Sharing Breast Milk

Four hundred and nine (409) respondents said they were indeed concerned about sharing their breast milk with other mothers. They gave various reasons in support of their decision not to share their breast milk. Two main themes emerged from their responses; these were, “Whether it is a healthy practice” and “African beliefs”.

1. Concern about Breast Milk Sharing Being a Healthy Practice

Respondents expressed various concerns and questions pertaining to health and safety issues associated with sharing their breast milk. The excerpts cited below shed more light on participant concerns:

Excerpt 17
Is everyone’s breast milk good for newborn babies?
(Currently unemployed, 25 years, resident in Southern part of Ghana).
Excerpt 18
Is it safe? Is it a healthy practice?
(Public sector worker, above 33 years, resident in Southern part of Ghana).

2. African Belief System

Some of the respondent indicated that they had concerns about sharing their breast milk due to their African beliefs. Excerpts 19 and 20 below illustrate participant thoughts about cultural mores associated with their African belief and value system.

Excerpt 19
As Africans and Ghanaians, we hold lots of sentiments regarding feeding babies with other woman’s body fluids.
(Public sector worker, above 35 years, resident in Southern part of Ghana).
Excerpt 20
Looking at the way Ghanaians are superstitious; I don’t think parents or relatives will allow their baby share breast milk with anyone.
(Public sector worker, 31 years, resident in Southern part of Ghana).

F. Not Concerned about Sharing Breast Milk

Six hundred and forty-one (641) respondents indicated that they were not concerned about sharing their breast milk with other mothers. Responses are categorized into two themes namely: “Breast milk is good” and “Help to a child”. Each theme is exemplified and explicated below.

1. Goodness and Suitability of Breast Milk for Children

Most of the respondents indicated that they had no concerns with sharing breast milk because they viewed breast milk as being good and being essential for every child’s nutritional needs. Two respondents shared the following:

Excerpt 21
Breast milk is good for every child so why should I be worried about sharing. I am willing to share if only the other mother accepts.
(Currently unemployed, above 35 years, resident in Southern part of Ghana).
Excerpt 22
I do not mind so long as I can ascertain that it is being used for a good cause. I have a cousin whose mother died at childbirth. I remember that one of her aunts breastfed her...If it would give a child a good start in life, why not?
(Private sector worker, above 35 years, resident in Southern part of Ghana).

2. Breast Milk’s ‘Help’ or Nutritional Benefit to Babies

Still acknowledging the benefits that a child stands to gain from breast milk, the respondents indicated that they did not have concerns as long as they knew that the donated milk would be helpful to the child. Two excerpts (23 and 24) are cited for exemplification and elucidation.

Excerpt 23
Because it will help a baby who really needs it. (Private sector worker, 34 years, resident in Southern part of Ghana).
Excerpt 24
I think is a humanitarian duty. (Public sector worker, 34 years, resident in Southern part of Ghana).

## 4. Discussion

Our research shows that out of the 1050 women who participated in the study, 853 (81%) indicated they breastfed their children, only 25 (3%) reported they did not breastfeed their children, and 172 (16%) did not have children. Our results indicate that breastfeeding is high among the respondents for this study compared to results from Ghana in general [5]. This high participation in breastfeeding may be due to the *Child Health Program* of the Ghana Health Service (GHS), an intervention that aims to promote exclusive breastfeeding in Ghana [3].

A majority of the respondents who provided responses for why they breastfed their children referred to the nutritional benefits of breast milk and the relative cost-effectiveness of breastfeeding. Our findings are supported by evidence from Chowdhury et al. (2015) and Eidelman et al. (2012) that state positive results relating to the nutritional benefits of human milk [17,18]. In our study, 3% of participants reported that they did not breastfeed their child(ren), citing insufficient breastmilk supply as the main reason for the inability to breastfeed. While our study did not explore exclusive breastfeeding rates in our sample, insufficient breastmilk supply is reported as a major hindrance to breastfeeding in general among Ghanaian women. For instance, a qualitative study by Otoo, Lartey & Pérez-Escamilla (2009) conducted with 35 women residing in the Eastern region of Ghana report mother’s perceived insufficient breastmilk supply as a barrier to exclusive breastfeeding [19]. In addition to this challenge, some mothers have also reported breast and nipple pain brought on by poor latching and improper positioning of the baby, which affects the mother’s desire and ability to breastfeed [19]. Consequently, there is a need to strengthen and improve the lactation education offered to expectant and postpartum women during antenatal and postnatal visits. This will give mothers the necessary tools to increase their breastmilk supply, thereby preventing women from resorting to informal milk sharing practices.

In this study, respondents point to the nutritional efficacy of breastmilk as the reason for their willingness to formally donate extra milk; however, some participants would only offer their milk contingent upon the willingness of a mother’s acceptance of the milk donation for her infant. This finding is quite significant as it underscores the need for further education about milk donation. Further, we suggest that health promotion efforts aimed at establishing formal and safer approaches to breastmilk sharing and donation, in the form of human milk banks, must invest in targeted health education campaigns that teach about the benefits, essence and principle of the donated breast milk to pregnant and lactating Ghanaian women.

In our sample, 8% of women reported ever sharing breastmilk with another mother. Indeed, the informal sharing of milk, commonly referred to as wet nursing, is not a new concept. Prior studies affirm that wet nursing has been practiced in various cultures for decades [20,21,22]. Existing studies shed light on the scope of wet nursing and milk sharing in Sub-Saharan African countries. For instance, Kimani-Murage et al. (2019) in their study found that among the 868 mothers employed in the study, 1% of the sample reported sharing breastmilk directly with another mother, while about 13% of the women reported seeing or hearing about the practice within their communities [23]. Again, participants in a study conducted by Coutsoudis et al. (2011) affirmed the existence of wet nursing and milk sharing in their respective communities prior to the emergence of HIV epidemic in the country [24]. In a Zimbabwean study of 535 health workers, 67% reported having knowledge of wet nursing [25]; In West Africa specifically, evidence of wet nursing has been reported in Nigeria [26], Ivory Coast [27] and in Burkina Faso [28]. Although the aforementioned studies provide some evidence of the existence of milk sharing and wet-nursing practice in Sub-Sharan Africa, our Ghanaian sample reported a higher percentage of milk sharing relative to that which is reported in other African nations. Hence, findings of this study may not be generalized to similar populations. Nonetheless, this study opens up the discussion to investigate further the practice of milk sharing in Sub-Sharan African countries; Results will aim to leverage the practice for the development of safer alternatives by establishing Human milk banks that feed low birthweight and preterm infants as well as children whose mothers experience delayed lactation or insufficient supply. This will help to reduce neonatal mortality in the region.

In our study, respondents who specified that they were worried about sharing their breastmilk attributed their concern to the safety of the milk. Specifically, respondents were concerned about the risk of disease transmission through another mother’s breastmilk. These concerns are warranted as prior research documents several hazards of sharing breastmilk including the risk of transmitting infectious diseases like HIV, tuberculosis, prescription medication and other illicit substances used by the donor that may be harmful to the newborn [3,29]. Other hazards include environmental contaminants that may be transported through unhygienic storage and handling of unprocessed milk [30]. Additionally, breastmilk that is repeatedly frozen and thawed may lose some of its essential bioactive factors [31]. With this in mind, it is imperative that Ghanaian mothers be educated on the risks inherent in milk sharing and to be informed about safer channels of milk donation.

### 4.1. Limitations

This study is not without limitations. First, some participants failed to answer the open-ended questions. Secondly, although we pilot tested the research questions, it is possible that some study participants may have misunderstood some survey questions. Although English is the official Language in Ghana, several people speak it as a second language which may impact complete comprehension of study questions. Nevertheless, the study recruited participants from various regions of Ghana and thus has strong representation of prevailing views of Ghanaian women with regard to breastfeeding and milk sharing. Last, this study was conducted in a single African country Ghana, which limits the generalizability of our findings to other Sub-Saharan African countries.

### 4.2. Recommendations and Future Research

Based on concerns raised by participants about the safety of donated breastmilk, there is the need to develop targeted education for Ghanaian women. Our recommendation is to incorporate milk banking education as part of existing breastfeeding campaigns. In order to allay the fears of potential breastmilk donors, human milk banks must be certified and regulated by Food and Drugs Authority to instill confidence in the milk screening processes. The Ghana FDA is a trusted entity within the community whose regulatory and supervisory role will ensure that breastmilk collection agencies that are established in the future adhere strictly to screening protocols just as in the case of Blood Bank services in the country.

Again, concerns about milk safety underline the need for education about the hazards of milk sharing/ wet nursing. Women must be educated on safer approaches to milk sharing through human milk banks (HMB). Future studies should explore adequate training and education of health professionals as prior studies discuss the key role of healthcare providers in shaping mother’s perceptions, attitudes and beliefs about HMB [32]. Additionally, future studies may examine effective health communication strategies that will have the best impact on promoting safer alternatives to milk sharing among Ghanaian women.

Further, subsequent studies may also explore socio-cultural factors, beliefs, and superstitions from the viewpoint of significant family members (grandmothers, fathers, mothers-in-law) and traditional authorities that are likely to pose obstacles for potential breastmilk donors. Further studies are warranted to assess opinions of health professionals regarding the feasibility and readiness of establishing donor breastmilk banks in Ghana. Information about logistics and resource constraints, handling and any anticipated storage and preservation challenges toward the establishment of breastmilk banks are also needed. This assessment is crucial in informing stakeholders and policymakers of gaps that need to be addressed in order to provide safer channels for milk donation.

Moreover, future studies could consider the possibility of advancing evidence on the impact of receiving donor breast milk relative to feeding with infant formula on newborn outcomes through robust randomized-controlled trials.

## 5. Conclusions

In sum, this current study provides evidence of relatively high self-reported breastfeeding rates among Ghanaian women. While majority of study participants (81%) indicated that they breastfed their children, a small but significant number of women (3%) reported that they did not breastfeed their child, attributing their inability to insufficient breastmilk supply. This underscores the need to improve lactation education and counselling to expectant and postpartum Ghanaian women. Again, evidence of informal milk sharing practices among our participants call for the implementation of safer alternatives to milk sharing. To this effect, our study findings indicate that a majority of Ghanaian women do not have concerns about sharing their milk; while this is a good indicator of potential milk donation, there is the need to establish formal and safer channels of breastmilk donation through a Human Milk Banking system. Hence, targeted education for pregnant and lactating women about the benefits of donating excess milk through safer channels for feeding vulnerable infants are warranted. Concerns raised by study participants about the safety of shared milk are indeed justified; Ghanaian women must be educated on the risks and dangers of sharing breastmilk directly to another mother. Thus, breastfeeding and the use of certified and safe donor’s human milk will improve newborn outcomes and help save the lives of Ghana’s most vulnerable infants.

## Figures and Tables

**Table 1:** Socio-Demographic Characteristics of Respondents *(n* = 1050).

	Frequency (F)	Percentage (%)
Country of Origin		
Ghanaian	1050	100
Residence		
Southern Part of Ghana	921	87
Northern Part of Ghana	121	12
Outside Ghana (Diaspora)	8	1
Age		
Below 35	748	71
Above 35	302	29
Occupation		
Public Sector	708	68
Private sector	287	27
Currently Unemployed	55	5
Educational level		
Junior High school or less	140	13
Senior High School	137	13
Some College	129	12
Bachelor’s Degree	418	40
Master’s Degree	174	16
Some Doctoral Level Courses	8	1
Doctoral Degree	16	2
No Formal Education	28	3

Note: Public sector refers to employment in an organization owned and controlled by the government. e.g., teacher, nurse etc. Private sector refers to employment in an organization owned and controlled by individuals. e.g., trader, plumber, etc.

## Data Availability

The study data are available upon request from the corresponding author, subject to appropriate ethical review by the IRB of the author’s institution.

## Appendix A

**Table A1:** Summary of study questions, themes, subthemes and participant quotes.

Research Question	Themes and Subthemes	Participant Quotes
Why mothers breastfeed their children	Breastmilk being ideal for child development	Breast milk is very vital and ideal for child development. It contains all the nutrients babies need to grow
		Because it helps in good child development and also prevent infections.
	Breastmilk being affordable	Because it is cheaper and a better option
		Breast milk contains the right nutrients for babies, and it is cheap and readily available
Why Mothers Chose Not to Breastfeed Their Children	Insufficient Breastmilk	I did not have sufficient breast milk
Motivation for Sharing Breast Milk with Other Women	Insufficient Breast Milk Production by Infants’ Mothers	My baby refused to suckle, and the other woman saw me expressing and discarding my breast milk so she came, pleading to give it to her instead because she had inverted nipples and also couldn’t produce breast milk and her baby was reducing in weight because she could not afford formula
		I was engorged. And she wasn’t producing enough to feed her child but was determined to stick to the 6 months rule.
	Mothers Unavailability Prompting mothers to share their Breast Milk	The baby was same month as mine. His mother had to go somewhere and return quickly but something delayed her, and baby started crying so I breastfed him.
		Her breast milk was initially not flowing and later [the mother] was admitted at the hospital because of complications.
Motivation for Not Sharing Breast Milk with Other Women	Absence or Lack of Opportunity to Share Breast Milk	No opportunity to do so.
		I produce enough and haven’t had the opportunity to share.
	Not Screened	Well, I know that one need to be screen before and certified before sharing her breast milk to other mothers and I, so I believe am not fit until I get screened
		Because no screening has been done and we know breast milk can transfer infections.
	Insufficient supply of Breast Milk	Unfortunate I did not have enough breast milk myself, aside that I do not know any other breastfeeding mom who was in dire need of breast milk.
		I seldom have enough to spare.
	Personal Perceptions about Breast Milk Sharing	Because only my baby needs my breast milk, and I don’t think someone in Ghana will allow her baby to be fed by a person who isn’t even a relative.
		Because they feel some way when you offer it [breast milk] to them… I have offered it before, but I was turned down.
Concerned about Sharing Breast Milk	Concern about Breast Milk Sharing Being a Healthy Practice	Is everyone’s breast milk good for newborn babies?
		Is it safe? Is it a healthy practice?
	African Belief System	As Africans and Ghanaians, we hold lots of sentiments regarding feeding babies with other woman’s body fluids.
		Looking at the way Ghanaians are superstitious; I don’t think parents or relatives will allow their baby share breast milk with anyone.
Not Concerned about Sharing Breast Milk	Goodness and Suitability of Breast Milk for Children	Breast milk is good for every child so why should I be worried about sharing. I am willing to share if only the other mother accepts.
		I do not mind so long as I can ascertain that it is being used for a good cause. I have a cousin whose mother died at childbirth. I remember that one of her aunts breastfed her...If it would give a child a good start in life, why not?
	Breast Milk’s ‘Help’ or Nutritional Benefit to Babies	Because it will help a baby who really needs it
		I think is a humanitarian duty.
